# Rescue therapy with rifabutin regimen for refractory *Helicobacter pylori* infection with dual drug-resistant strains

**DOI:** 10.1186/s12876-020-01370-4

**Published:** 2020-07-10

**Authors:** Chia-Jung Kuo, Cheng-Yu Lin, Puo-Hsien Le, Pi-Yueh Chang, Chih-Ho Lai, Wey-Ran Lin, Ming-Ling Chang, Jun-Te Hsu, Hao-Tsai Cheng, Chi-Nan Tseng, Chun-Jung Lin, Ming-Yao Su, Sen-Yung Hsieh, Cheng-Tang Chiu

**Affiliations:** 1 Department of Gastroenterology and Hepatology, Chang Gung Memorial Hospital at Linkou, 5, Fushin Street, Kweishan, Taoyuan, Taiwan, Republic of China 333; 2 Chang Gung University, College of Medicine, Taoyuan, Taiwan; 3 Department of Laboratory Medicine, Chang-Gung Memorial Hospital, Taoyuan, Taiwan; 4 Department of Microbiology and Immunology, Chang Gung University, Taoyuan, Taiwan; 5 Molecular Infectious Disease Research Center, Department of Pediatrics, Chang Gung Memorial Hospital, Linkou, Taiwan; 6 Department of Surgery, Chang Gung Memorial Hospital at Linkou, Taoyuan, Taiwan; 7 Department of Gastroenterology and Hepatology, New Taipei Municipal TuCheng Hospital (Built and Operated by Change Gung Medical Foundation), Taipei, Taiwan; 8 Department of Cardiothoracic and Vascular Surgery, Chang Gung Memorial Hospital, Taoyuan, Taiwan; 9 Department of Molecular Medicine and Surgery, Karolinska University Hospital, Karolinska Institutet, Stockholm, Sweden

## Abstract

**Background:**

There is no current standard rescue treatment for dual drug-resistant strains of *Helicobacter pylori (H. pylori)*. This aim of this study was to investigate the efficacy of rifabutin-based triple therapy for patients infected with dual drug-resistant strains to clarithromycin and levofloxacin.

**Methods:**

After 2 or 3 *H. pylori* treatment failures, patients underwent upper endoscopy with tissue biopsies. Phenotypic and genotypic resistances were determined using agar dilution test and polymerase chain reaction with direct sequencing, respectively. Patients infected with dual drug-resistant (clarithromycin and levofloxacin) strains and receiving rifabutin-based triple therapy (rifabutin 150 mg bid, amoxicillin 1 g bid and esomeprazole 40 mg bid for 10 days) were enrolled. Eradication status was determined by 13C-urea breath test 4 weeks after treatment completion.

**Results:**

A total of 39 patients infected with dual drug-resistant strains were enrolled in this study, with a mean age of 55.9 years. The eradication rate was 79.5% (31/39) (95% confidence intervals: 54.96% ~ 111.40%). Adverse event was reported in 23.1% (9/39) of patients but they were mild and tolerable. In univariate analysis, no factor was identified as an independent predictor of eradication failure.

**Conclusions:**

Our current study demonstrated that rifabutin-based triple therapy was well tolerated and yielded an acceptable eradication rate for patients infected with dual drug-resistant strains of *H. pylori*.

## Background

*H. pylori* is a well-known pathogen associated with several upper gastrointestinal diseases, including peptic ulcer disease, atrophic gastritis and malignancies (gastric cancer and mucosa-associated lymphoid tissue lymphoma) [1, 2]. In areas of low clarithromycin resistance, clarithromycin-based triple therapy is recommended as first-line empirical treatment [3]. The successful rate of eradication of *H. pylori* have declined recently, mainly due to the increasing prevalence of drug resistance [4, 5]. In regions with high resistance to clarithromycin, the effectiveness of the standard triple therapy is lower than 80% [6]. Levofloxacin-based triple therapy (proton pump inhibitor [PPI], amoxicillin, and levofloxacin) is considered a rescue treatment if one or more prior treatment attempts failed. However, resistance to fluoroquinolones is also emerging, and the prevalence in Europe is now close to 15% [7].

The Maastricht IV/Florence consensus report suggests that culture and antimicrobial sensitivity testing should be performed when designing a treatment strategy after one or two treatment failures with different antibiotics [3].

Rifabutin, which mostly used against *Mycobacterium tuberculosis* and *Mycobacterium avium intracellulare* infection, has been applied as an alternative regimen for *H. pylori* eradication [8–10]. The aim of the present study was to evaluate the efficacy of rifabutin regimen in patients infected with *H. pylori* dual resistant to clarithromycin and levofloxacin.

## Methods

### Study design

**Fig. 1:**
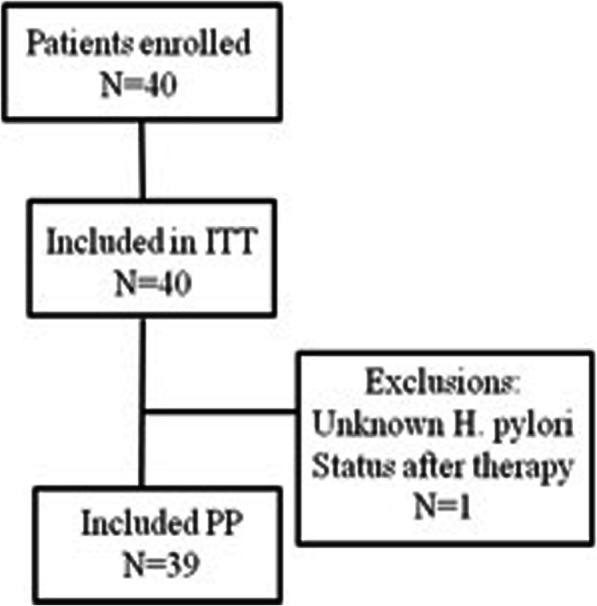
Schematic flow chart

After two or three times *H. pylori* eradication failure, patients underwent upper gastrointestinal endoscopy with biopsy of the stomach mucosa for subsequent bacterial culture and molecular analysis for drug resistance. Phenotypic and genotypic resistance was determined using agar dilution test and polymerase chain reaction (PCR) with direct sequencing, respectively. Only patients infected with *H. pylori* strains harboring dual resistance to clarithromycin and levofloxacin but sensitive to amoxicillin were enrolled in this study and treated with rifabutin-based therapy (rifabutin 150 mg bid, amoxicillin 1 g bid and esomeprazole 40 mg bid) for 10 days. Eradication status was determined by 13C-urea breath test performed 4 weeks later after treatment completion. Self-reported drug adherence and adverse events were recorded during follow-up visiting. Figure 1 demonstrates the schematic flow chart of the study design. This study was approved by the Institutional Review Board of Chang Gung Memorial Hospital (IRB No. 201701000A3).

The primary end point of the study was eradication rate. The secondary end point was the rate of adverse effects. Associated factors for successful eradication were also assessed. Patients who lost 13C-urea breath test follow-up and who with unsuccessful *H. pylori* culture were excluded from this study.

### Antibiotic susceptibility

The specimens of gastric biopsy were incubated at 37 °C under microaerophilic conditions for 10–14 days. Positive cultures were usually identifiable after 3 to 5 days of incubation. Isolates were identified as *H. pylori* according to colony morphology, Gram staining, and the results of urease, catalase, and oxidase tests.

Antibiotic susceptibility was determined by agar dilution test (E-test) of *H. pylori* culture and real-time PCR for DNA sequencing of gastric biopsy specimens. Isolated *H. pylori* strains were analyzed for amoxicillin, clarithromycin, and levofloxacin resistance using break points for minimum inhibitory concentrations of ≥0.5, ≥1, and > 1 μg/mL, respectively. Point mutations (A2143G, A2142G, and A2142C) in the 23S rRNA gene and point mutations in the DNA gyrase A gene (codons 87 and 91), which associated with clarithromycin and levofloxacin resistance respectively, were also determined.

### Statistical analysis

Comparison of the patients’ demographic characteristics, eradication rates, and frequency of adverse events was conducted using Fisher’s exact test and Student’s t-test, as appropriate. Statistical analyses were performed using SPSS version 22 for Windows (SPSS Inc., Chicago, IL, USA). Data are expressed as mean ± standard deviation.

## Results

### Baseline demographic and clinical data

A total of 39 patients (males, 16; female, 23; mean age, 55.9 ± 12 years) infected with dual drug-resistant *H. pylori* strains (clarithromycin and levofloxacin) were enrolled for further analysis. The indications for eradication therapy were noulcer dyspepsia (79.5%) and peptic ulcer disease (20.5%), including six cases of gastric ulcer and two cases of duodenal ulcers. Five cases were active smoker.

### Eradication rates

Eradication status was determined by ^13^C-urea breath test, which was carried out no earlier than 4 weeks and up to 8 weeks after cessation of treatment. The cut-off value for a negative UBT was < 4. Overall, the infection was eradicated in 31 patients, corresponding to an eradication rate of 79.5% (95% confidence intervals: 54.96% ~ 111.40%). Age (*p* = 0.45), gender (*p* = 0.56) or smoking (*p* = 0.98) was not associated with eradication failure by using the univariate analysis.

### Adverse effects

Among the 39 patients who receiving rifabutin-based triple therapy, there were only mild and tolerable adverse events reported, including anorexia (12.8%), constipation (5.1%), skin itchiness (2.6%) and diarrhea (2.6%). None of the patients stopped therapy due to side effects.

## Discussion

The possible causes for eradication therapy failures include antibiotic resistance, smoking, high bacterial load before treatment, bacterial genotype, poor patient compliance, and polymorphisms of metabolism of PPIs. With the increasing prevalence of antimicrobial resistance, the eradication rate of *H. pylori* has been declined. The Maastricht IV/Florence consensus report recommends that culture and antimicrobial sensitivity testing should be performed after one or two treatment failures with different antibiotics [3]. Meanwhile, according to the Maastricht V/Florence consensus report, after the first failure, if endoscopy is carried out, culture and standard antimicrobial susceptibility testing are recommended to tailor the treatment [11].

The prevalence of *H. pylori* strains that resistant to more than one antibiotic was 15% in the United States and 8.9% in Europe [12]. According to the study of Liou et al. [13], the secondary resistant rates of clarithromycin, levofloxacin, and metronidazole were as high as 92.5, 70.1, and 87.7%, respectively, in patients who had received these antibiotics in their prior therapies in Taiwan.

When selecting salvage therapy, previously used antibiotics should be avoided. The use of a salvage regimen for patients with persistent *H. pylori* infection is an increasingly common scenario but remains a challenge for clinicians because only a few antibiotics are available. Currently, a standard salvage regimen is still lacking. Our data showed that 10 days rifabutin-based triple therapy was well tolerated and yielded an acceptable *H. pylori* eradication rate for patients infected with dual drug-resistant strains to clarithromycin and levofloxacin.

Rifabutin inhibits the beta-subunit of DNA-dependent RNA polymerase of *H. pylori*, which is encoded by the *rpoB* gene. Rifabutin-based triple therapy has been applied as a rescue treatment. A low rate of resistance (0.24%) to rifabutin was noted in *H. pylori* strains isolated from 414 Japanese patients. The only rifabutin-resistant strain detected showed a point mutation in the *rpoB* gene and was isolated from a patient with a history of rifampin treatment for pulmonary tuberculosis. The mean *H. pylori* rifabutin resistance rate (calculated from 11 studies, including 2982 patients) was 1.3% (95% confidence interval [CI], 0.9–1.7%) [14]. The respective cure rates for second-line (223 patients), third-line (342 patients), and fourth−/fifth-line (95 patients) rifabutin therapies were 79% (95% CI, 67–92%), 66% (95% CI, 55–77%), and 70% (95% CI, 60–79%), respectively [14].

The American College of Gastroenterology clinical guideline suggests a rifabutin-based triple regimen consisting of a PPI, amoxicillin, and rifabutin for 10 days as a suggested salvage regimen, but it has a very low quality of evidence for duration [15]. However, the ideal length of treatment for the rifabutin regimen remains unclear. Van Zanten et al., report that PPI twice daily, amoxicillin 1 g twice daily and rifabutin 300 mg once daily for 1 week was prescribed in 16 patients for rescue therapy and the success rate was 63% [16]. In some reports, a 7-day course has been equally effective as the 10- to 14-day regimens, whereas others have found that this shorter duration dramatically reduced the efficacy in terms of eradication rates. High-dose PPI seems to play some role. A previous study in Korea study demonstrated that higher eradication rate was achieved when double doses (lansoprazole 60 mg bid) were administered instead of standard doses (lansoprazole 30 mg bid) with the same rifabutin-amoxicillin combination (intention-to-treat, 96.3% vs 78.1%. *p* = 0.51) [17].

A recent study by Fiorini et al. [18] reported that the efficacy of the 12-day rifabutin-based triple therapy (with esomeprazole 40 mg bid, amoxicillin 1 g bid, and rifabutin 150 mg od) for patients infected with multidrug-resistant strains (clarithromycin, metronidazole, and levofloxacin) was 82.9% (95% CI, 78.3–87.5) by intention-to-treat analysis and 88.7% (95% CI, 84.7–92.7) at per-protocol analysis. The mean rate of adverse effects was 22% (19–25%). A long-term prospective study in a large cohort with 302 difficult-to-treat patients revealed that rifabutin 150 mg, amoxicillin 1 g and a standard dose of proton pump inhibitor, twice daily for 14 days achieved eradication rate in 72.7% (per-protocol) and 71.5% (intention-to-treat) respectively. A univariate analysis showed that gender, ethnic background, smoking habits and familial history of gastric diseases were not predictive factors of response [19]. Except combining with amoxicillin and PPI, a quadruple combination with rifabutin, bismuth, minocycline and rabeprazole had been reported to achieve 77.7% eradication rate but only 21 patients were enrolled [20]. The efficacy of rifabutin treatment is summarized in Table 1.

**Table 1:** Summary of outcomes of rifabutin based triple therapy in *H. pylori* infection

Author(s) and year	Country	Drugs and doses	Duration of treatment (days)	No. of patients	No. of previously failed treatment	Eradication rate (%)
Fiorini G 2018 [18]	Italy.	esomeprazole 40 mg bid, amoxicillin 1 g bid, and rifabutin 150 mg od	12	254	2	82.9% intention-to-treat
Ribaldone DG 2019 [19]	Italy.	Rifabutin 150 mg bid Amoxicillin 1 g bid PPI bid	14	302	2	71.5% intention-to-treat
Van Zanten et al. 2010 [16]	Canada	Rifabutin 300 mg od Amoxicillin 1 g bid PPI bid	7	16	3	63%
Lim et al. 2014 [17]	Korea	Rifabutin 150 mg bid Amoxicillin 1 g tid Lansoprazole 60 mg bid	7	27	2	96.3 intention-to-treat
Lim et al. 2014 [17]	Korea	Rifabutin 150 mg bid Amoxicillin 1 g tid Lansoprazole 30 mg bid	7	32	2	78.1 intention-to-treat
Ierardi et al. 2014 [20]	Italy	Rifabutin 150 mg bid Minocycline 100 mg bid Bismuth 120 mg qid Rabeprazole 20 mg bid	10	21	2	77.7%

One significant concerning in rifabutin treating was adverse effects of myelotoxicity. Lower doses and/or a shorter duration would lower the possibility of myelotoxicity. In the present study, we used rifabutin 150 mg bid, amoxicillin 1 g bid and esomeprazole 40 mg bid for 10 days and no neutropenia was observed.

Our study has some limitations. First, it was a single-centered study and the sample size was not large. There was approximately 5–10% of patients fail to eradicate *H. pylori* infection after commonly used Clarithromycin- and Levofloxacin-based therapy. However, in recent literatures focus on *H. pylori* eradication with rifabutin-based triple therapy (as Table), the case numbers ranged from 16 to 302, and our sample size could be an acceptable one. Second, our study lack of randomization to different rescue regimen. For example, a larger, multicenter study comparing this treatment with a bismuth quadric-therapy will be much more helpful. Third, there is some concern about a wide-spread use of rifabutin for *H. pylori* eradication. Rifabutin has been used as an antimycobacterial drug and indications for the drug should be chosen very carefully to avoid further development of resistance. Finally, it needs long-term follow up for those patients who were failed of rifabutin-based *H. pylori* eradication.

## Conclusions

Our current study demonstrated that 10 days rifabutin-based triple therapy was well tolerated and yielded an acceptable eradication rate for patients infected with dual drug-resistant *H. pylori*.

## Data Availability

The datasets used and analysed during the current study are available from the corresponding author on reasonable request.

## Abbreviations

*H. pylori*: *Helicobacter pylori*
PPI: Proton pump inhibitor

## References

[1] McColl KE (2010). Clinical practice. Helicobacter pylori infection. N Engl J Med.

[2] Lee YC, Chiang TH, Chou CK, Tu YK, Liao WC, Wu MS, Graham DY (2016). Association between helicobacter pylori eradication and gastric cancer incidence: a systematic review and meta-analysis. Gastroenterology.

[3] Malfertheiner P, Megraud F, O’Morain CA, Atherton J, Axon AT, Bazzoli F, Gensini GF, Gisbert JP, Graham DY, Rokkas T, El-Omar EM, Kuipers EJ (2012). The European helicobacter study group (EHSG): Management of Helicobacter pylori infection—the Maastricht IV/Florence consensus report. Gut.

[4] Megraud F (2004). H pylori antibiotic resistance: prevalence, importance, and advances in testing. Gut.

[5] Megraud F (2004). Basis for the management of drug-resistant helicobacter pylori infection. Drugs.

[6] Graham DY, Fischbach L (2010). Helicobacter pylori treatment in the era of increasing antibiotic resistance. Gut.

[7] Gisbert JP, Morena F (2006). Systematic review and meta-analysis: levofloxacin-based rescue regimens after helicobacter pylori treatment failure. Aliment Pharmacol Ther.

[8] Maddix DS, Tallian KB, Mead PS (1994). Rifabutin: a review with emphasis on its role in the prevention of disseminated Mycobacterium avium complex infection. Ann Pharmacother.

[9] Megraud F, Lamouliatte H (2003). Review article: the treatment of refractory helicobacter pylori infection. Aliment Pharmacol Ther.

[10] Bock H, Koop H, Lehn N, Heep M (2000). Rifabutin-based triple therapy after failure of helicobacter pylori eradication treatment: preliminary experience. J Clin Gastroenterol.

[11] Malfertheiner P, Megraud F, O’Morain CA, Gisbert JP, Kuipers EJ, Axon AT, Bazzoli F, Gasbarrini A, Atherton J, Graham DY, Hunt R, Moayyedi P, Rokkas T, Rugge M, Selgrad M, Suerbaum S, Sugano K, El-Omar EM (2017). European helicobacter and microbiota study group and consensus panel: Management of Helicobacter pylori infection—the Maastricht V/Florence consensus report. Gut.

[12] De Francesco V, Giorgio F, Hassan C, Manes G, Vannella L, Panella C, Ierardi E, Zullo A (2010). Worldwide H. pylori antibiotic resistance: a systematic review. J Gastrointest Liver Dis.

[13] Liou JM, Chen PY, Luo JC, Lee JY, Chen CC, Fang YJ, Yang TH, Chang CY, Bair MJ, Chen MJ, Hsu YC, Hsu WF, Chang CC, Lin JT, Shun CT, El-Omar EM, Wu MS (2018). Taiwan gastrointestinal disease and helicobacter consortium: efficacies of genotypic resistance-guided vs empirical therapy for refractory helicobacter pylori infection. Gastroenterology.

[14] Gisbert JP, Calvet X (2012). Review article: rifabutin in the treatment of refractory helicobacter pylori infection. Aliment Pharmacol Ther.

[15] Chey WD, Leontiadis GI, Howden CW, Moss SF (2017). ACG clinical guideline: treatment of Helicobacter pylori infection. Am J Gastroenterol.

[16] Van Zanten SV, Desai S, Best L, Cooper-Lesins G, Malatjalian D, Haldane D, Peltekian K (2010). Rescue therapy using a rifabutin-based regimen is effective for cure of helicobacter pylori infection. Can J Gastroenterol.

[17] Lim HC, Lee YJ, An B, Lee SW, Lee YC, Moon BS (2014). Rifabutin-based high-dose proton-pump inhibitor and amoxicillin triple regimen as the rescue treatment for helicobacter pylori. Helicobacter.

[18] Fiorini G, Zullo A, Vakil N, Saracino IM, Ricci C, Castelli V, Gatta L, Vaira D (2018). Rifabutin triple therapy is effective in patients with multidrug-resistant strains of helicobacter pylori. J Clin Gastroenterol.

[19] Ribaldone DG, Fagoonee S, Astegiano M, Durazzo M, Morgando A, Sprujevnik T, Giordanino C, Baronio M, De Angelis C, Saracco GM, Pellicano R (2019). Rifabutin-based rescue therapy for helicobacter pylori eradication: a long-term prospective study in a large cohort of difficult-to-treat patients. J Clin Med.

[20] Ierardi E, Giangaspero A, Losurdo G, Giorgio F, Amoruso A, De Francesco V, Di Leo A, Principi M (2014). Quadruple rescue therapy after first and second line failure for helicobacter pylori treatment: comparison between two tetracycline-based regimens. J Gastrointest Liver Dis.

